# Novel Biomarkers in Membranous Nephropathy

**DOI:** 10.3389/fimmu.2022.845767

**Published:** 2022-04-22

**Authors:** Qiuying Liu, Jianhua Liu, Baoxu Lin, Yue Zhang, Meichen Ma, Mei Yang, Xiaosong Qin

**Affiliations:** ^1^ Department of Laboratory Medicine, Shengjing Hospital of China Medical University, Shenyang, China; ^2^ Department of Laboratory Medicine, Beijing Haidian Hospital, Beijing Haidian Section of Peking University Third Hospital, Beijing, China

**Keywords:** membranous nephropathy, biomarker, phospholipase A2 receptor, proteome, metabolome, noncoding RNA, immune cell

## Abstract

Membranous nephropathy (MN) is the main cause of adult nephrotic syndrome (NS). The pathogenesis of MN is complex and involves subepithelial immune complex deposition. Approximately one-third of patients with MN develop end-stage renal disease (ESRD). Timely diagnosis and reasonable intervention are the keys to improving prognosis. In recent years, with the development of high-throughput technologies, such as mass spectrometry (MS), microarray, and sequencing technologies, the discovery of biomarkers for MN has become an important area of research. In this review, we summarize the significant progress in biomarker identification. For example, a variety of podocyte target antigens and their autoantibodies have been reported. Phospholipase A2 receptor (PLA2R) is the most well-established target antigen in MN. PLA2R and its autoantibodies have clinical significance, with both diagnostic and therapeutic value for MN. In addition, a variety of new biomarkers, including proteins, metabolites, noncoding RNAs (ncRNAs), and immune cells, have recently been found. These MN-related biomarkers have great significance in the diagnosis, progression, prognosis, and treatment response of MN.

## Introduction

**Figure 1:**
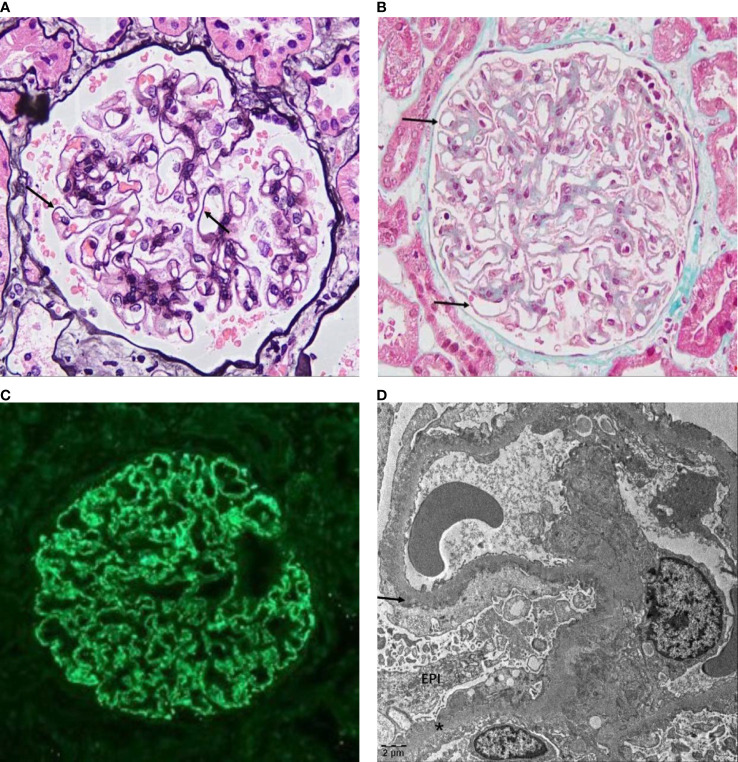
Pathological features of IMN. **(A)** Glomerulus from a patient with IMN showing the thickened GBM (arrow) under a light microscope stained with PASM (original magnification, 400×) and **(B)** pathognomonic antigen–antibody immune complex deposition on the epithelial side (arrow) stained with Masson (original magnification, 400×). **(C)** Immunofluorescence microscopy in IMN showed IgG (+++), fine granular deposition along capillary loops. **(D)** Electron micrograph of IMN showed that the GBM is irregularly thickened, the foot processes are diffusely fused, and more electron-dense deposits (arrow) can be seen under the epithelium (EPI) and in the basement membrane (*) (images were based on pathological data for renal biopsy cases in the Department of Nephrology of Shengjing Hospital).

Membranous nephropathy (MN) is the major cause of adult nephrotic syndrome(NS) and the second leading cause of primary glomerulonephritis and end-stage renal disease (ESRD). Approximately 80% of MN cases have no clear secondary causes, referred to as idiopathic membranous nephropathy (IMN) or primary membranous nephropathy (PMN), and approximately 20% of MN cases are secondary to autoimmune diseases, infections, malignancies, drug use, and heavy metal poisoning, referred to as secondary membranous nephropathy (SMN) (1). The pathological features of IMN are thickening of glomerular basement membrane(GBM) (**Figure 1A**) and antigen-antibody immune complex deposition in the subepithelial area of GBM (**Figure 1B**) under light microscopy. In addition, the glomerular capillary walls show bright granular staining of IgG deposition by immunofluorescence microscopy (**Figure 1C**), and electron-dense deposits in the GBM are visible by electron microscopy (**Figure 1D**).

**Figure 2:**
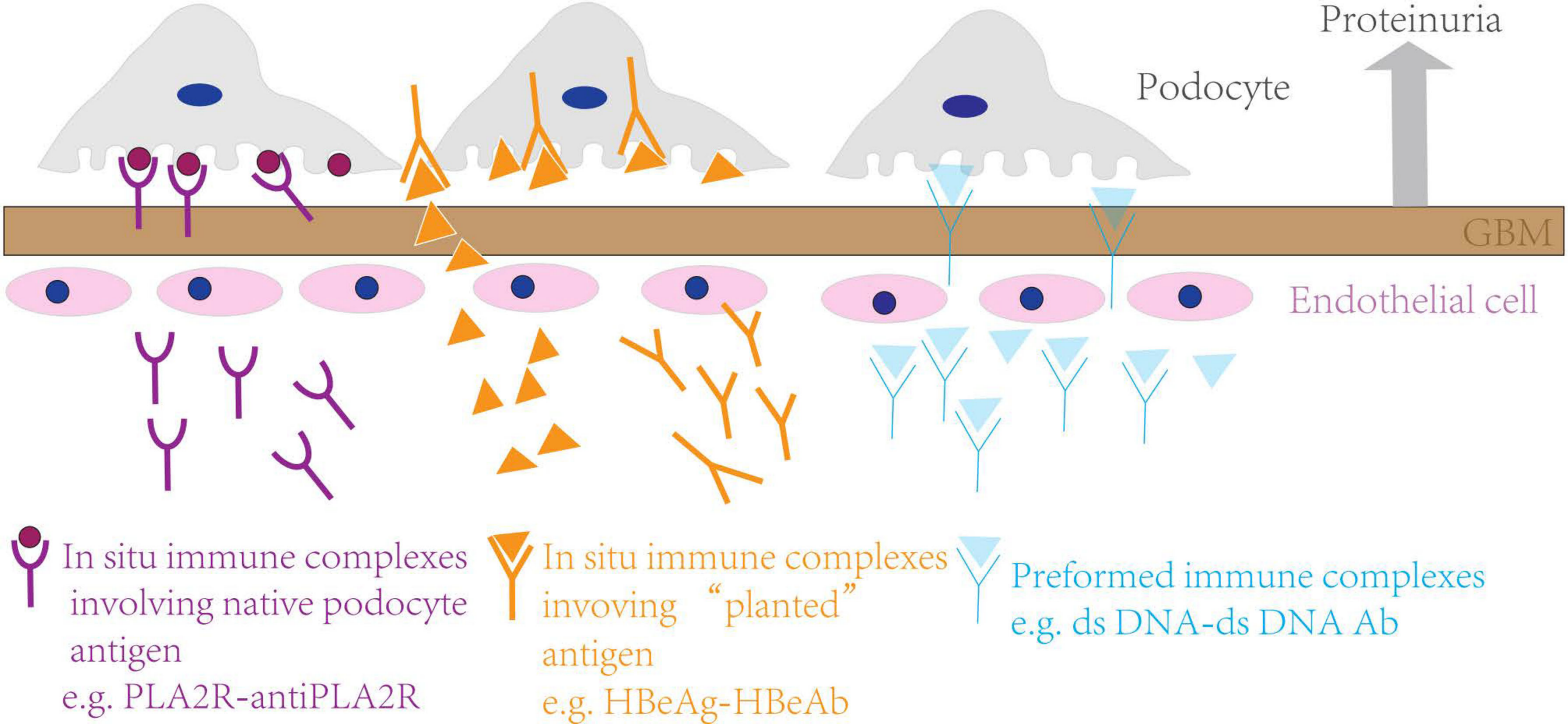
Pathogenesis of MN.

The pathogenesis of MN is complex, and the core process is the formation of an immune complex in the GBM. It is currently suggested that *in situ* immune complexes of circulating autoantibodies and their target antigens on glomerular podocytes are deposited under epithelial cells, resulting in complement activation, thereby destroying the podocyte structure and resulting in proteinuria (2). In addition, the deposition of preformed circulating antigens, such as viral, tumor, and thyroid antigens, under the epithelium that binds to corresponding antibodies to form an *in situ* immune complex and preformed circulating immune complexes, such as circulating double-stranded DNA (dsDNA)–double-stranded DNA antibody (dsDNA Ab) complexes in patients with systemic lupus erythematosus, under the epithelium may also be involved in the pathogenesis of MN (3–6) (**Figure 2**).

The clinical manifestations of MN are mainly NS and asymptomatic proteinuria. The disease course is characterized by a “one-third law”, in which approximately one-third of cases resolve spontaneously without treatment, one-third show persistent proteinuria, and one-third progress to ESRD in 5–10 years (7, 8).

At present, the gold standard for MN diagnosis is renal biopsy; however, owing to its invasive nature, its repeated application for clinical diagnosis is limited. Monitoring and the determination of curative effects in MN still largely depend on quantitative indicators of urinary protein and renal function; however, these indexes lack specificity. Therefore, it is of great clinical significance to explore sensitive, specific, and noninvasive biomarkers for the diagnosis and treatment of MN. In recent years, advances in high-throughput technologies have provided a basis for substantial breakthroughs in biomarker identification for MN diagnosis, disease monitoring, the judgement of treatment efficacy, prognostic evaluation, etc. In particular, we describe podocyte target antigens and autoantibody biomarkers, protein and metabolic markers, noncoding RNAs, and immune cell markers, with a focus on the detection technology and clinical value of various marker types.

## 1 MN-Related Podocyte Target Antigen and Autoantibody Biomarkers

The pathogenesis of MN involves complement-mediated podocyte injury caused by antibodies targeting podocyte antigens. Therefore, MN-related podocyte target antigens and their autoantibodies can provide reliable markers for diagnosis and monitoring.

**Figure 3:**
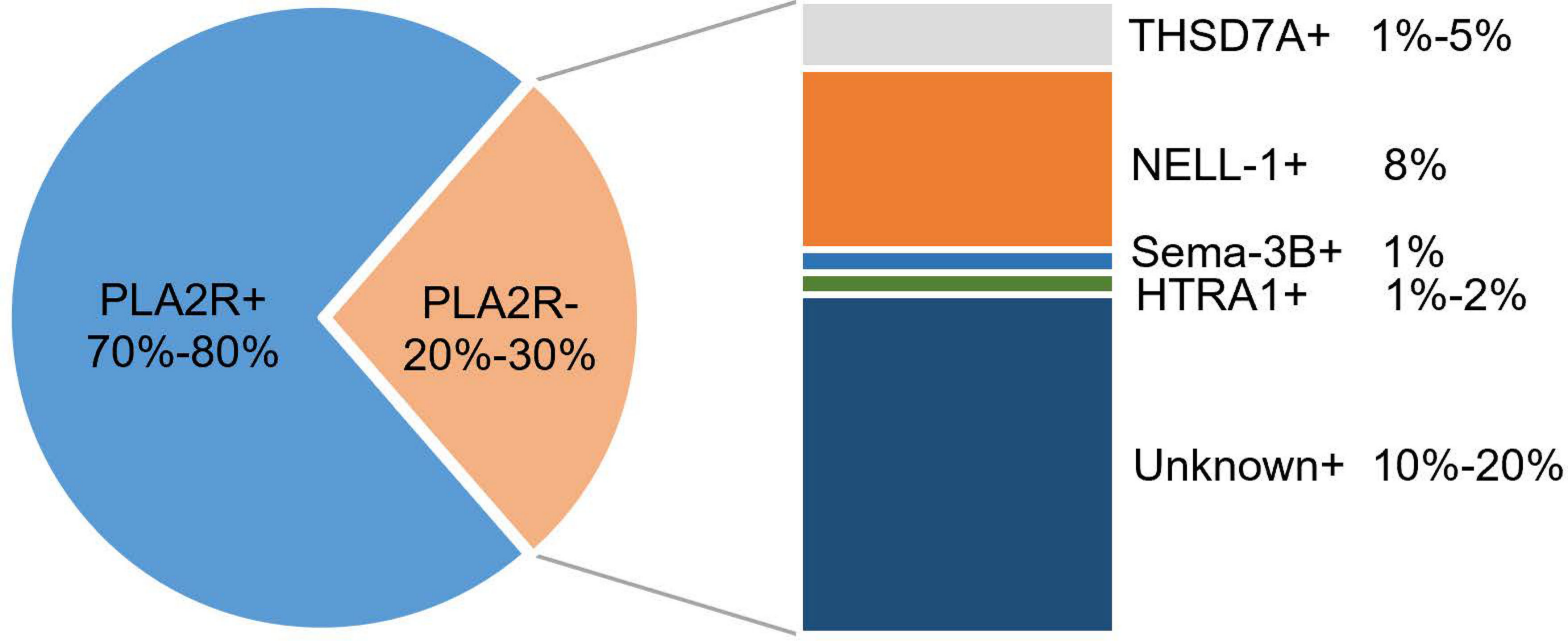
Distribution of podocyte antigens in patients with IMN.

Research aimed at the discovery of MN-related podocyte target antigens and their autoantibodies has continued for decades. A variety of human podocyte target antigens and their autoantibodies have been found. In 2002, Debiec et al. found that the autoantigen leading to neonatal MN is a neutral endopeptidase (NEP) in podocytes (9), and its autoantibodies are maternal alloantibodies caused by a hereditary NEP deficiency in the mother, rather than the antibodies produced by the child. It was not until 2009 that the first adult MN podocyte target antigen, M-type phospholipase A2 receptor (PLA2R), was detected in 70% of IMN serum and tissues by liquid chromatography with tandem mass spectrometry (LC-MS/MS) (10). Since the discovery of PLA2R, a variety of MN podocyte target antigens have been discovered by high-throughput MS. In 2014, Thomas et al. (11) identified another IMN podocyte target antigen, thrombospondin type-1 domain-containing 7A (THSD7A), in PLA2R-negative patients with IMN by MS. In 2019, Sethi et al. (12) identified exostatin/exostatin 2 (EXT1/EXT2) as a target antigen of MN in PLA2R-negative MN by laser microdissection and MS (LMD-MS). EXT1/EXT2 is the most common specific target antigen of PLA2R-negative MN and exists in SMN. In 2020, Sethi et al. (13) used LMD-MS to identify neural epidermal growth factor-like 1 (NELL-1) as another target antigen for PLA2R and THSD7A double negative patients. In the same year, LMD-MS was used to identify another target antigen, semaphorin-3b (Sema-3B), in the patients negative for PLA2R, THSD7A, EXT1/EXT2, and NELL-1 (14). In 2021, Caza et al. (15) identified neural cell adhesion molecule 1 (NCAM-1) as a new autoantigen of membranous lupus nephritis (LN) by LMD-MS. In the same year, high-temperature recombinant protein A1 (HTRA1) (16) and protocadherin 7 (PCDH7) (17) were identified as additional target antigens. Although the clinical characteristics of PCDH7 are not clear, it is speculated that it is an IMN target antigen. IMN-related target antigens can be detected in 80% of cases, of which approximately 70%–80% of IMN cases are PLA2R positive, approximately 1% are PLA2R and THSD7A double positive, and another 20%–30% are negative for PLA2R, including THSD7A, NELL-1, Sema3B, and THRA-related IMN (approximately 13%); however, 10%–20% of IMN-related target antigens remain to be discovered (1, 2, 16, 18–20) (**Figure 3**).

There are different predominant IgG subtypes in different podocyte target antigen-associated MN (**Table 1**). The majority of IgG subclasses of the earliest discovered autoantigens PLA2R- and THSD7A-associated IMN (approximately 80%–85% IMN) are IgG4, while EXT1/EXT2-related SMN (approximately 30%–40% SMN) is IgG1 (18–20). Therefore, in the majority of cases of MN, the dominant IgG subtypes in IMN and SMN are IgG4 and IgG1, respectively. However, it cannot accurately distinguish IMN from SMN, especially for the new antigens discovered in recent years. For example, the predominant autoantibody subtype in patients with NELL-1 and Sema-3B-related IMN is IgG1 (13, 14), and NEP, NCAM-1, and PCDH7 do not have the single majority IgG subtype (15, 17, 19). In addition, the main patient groups for different podocyte target antigen-related MN are different. Most of the patients with MN were adults, while the patients with NEP-related MN were mainly newborns, and Sema-3B-related MN patients were mainly children (**Table 1**).

**Table 1:** Summary of research progress on MN-related human podocyte target antigens.

Year	Antigen	Technique	IgG subtype	Patient	Clinical value	Reference
2002	NEP	Western blotting	IgG1, lgG4	Newborn baby	Diagnosis of IMN	(9)
2009	PLA2R	LC-MS/MS	IgG4	Adult	Diagnosis of IMN, Treatment response, Prognosis, Progression	(10)
2014	THSD7A	MS	IgG4	Adult	Diagnosis of IMN Treatment response, Prognosis	(11)
2019	EXT1/EXT2	LMD-MS	IgG1	Adult	Diagnosis of SMN	(12)
2020	NELL-1	LMD-MS	IgG1	Adult	Diagnosis of IMN	(13)
2020	Sema-3B	LMD-MS	IgG1	Children	Diagnosis of IMN	(14)
2021	NCAM-1	LMD-MS	IgG (inconsistent patten)	Adult	Diagnosis of MN	(15)
2021	HTRA1	LMD-MS	IgG4	Adult	Diagnosis of IMN	(16)
2021	PCDH7	LMD-MS	IgG1, IgG4	Adult	Diagnosis of MN	(17)

A large number of studies have proven that the above-mentioned MN-related podocyte antigens and autoantibodies can be used as biomarkers of MN and play auxiliary roles in the diagnosis, analyses of treatment responses, and prediction of prognosis. The clinical value of podocyte target antigen-based biomarkers (including autoantigens and their autoantibodies) of MN (**Table 1**) is described in the following sections.

### 1.1 PLA2R

Among the MN podocyte target antigen and autoantibody biomarkers found to date, PLA2R and anti- PLA2R antibody have better clinical diagnostic value for IMN than other biomarkers. A meta-analysis based on data from 10 studies involving 1,550 participants showed that in the active phase, the sensitivity and specificity of serum PLA2R antibody levels for the diagnosis of IMN are approximately 74.0% and 95.0%, respectively (21). Many studies have also shown that the sensitivity of anti-PLA2R antibodies for IMN diagnosis is >70%, and the specificity is close to 100% (22–24). Despite the high sensitivity and specificity of serum anti-PLA2R antibody levels for the diagnosis of IMN, there is no consensus on whether this approach can replace renal biopsy. Some scholars believe that renal pathology can provide more information than serum antibodies. In 2021, KDIGO guidelines indicated that patients with nephrotic syndrome (NS) and positive anti-PLA2R antibodies may not need renal biopsy for MN diagnosis. For patients with positive PLA2R antibodies, if there is an unexplained decrease in the estimated glomerular filtration rate (eGFR) or unresponsive to immunosuppressive therapy, renal biopsy is recommended (25).

Many studies have shown that PLA2R is more common in IMN than in SMN (26–31), suggesting that PLA2R has certain clinical value for distinguishing between the two types. However, it has also been detected in SMN (26–31), especially in hepatitis, sarcoidosis, and tumor-related SMN. Larsen et al. (31) detected PLA2R1 in 80 cases of SMN renal tissues by indirect immunofluorescence. PLA2R1 was positive in 64% of cases with hepatitis C (7/11) and in 75% of cases with sarcoidosis (3/4), 25% with tumors (3/12), and 2% with autoimmune diseases (1/46)-related SMN. Therefore, although PLA2R is more common in IMN, it cannot reliably distinguish IMN from SMN.

A number of studies have shown that PLA2R and its autoantibodies are closely related to the severity, treatment efficacy, and prognosis of MN. Levels of serum anti-PLA2R antibodies are related to serum albumin, serum creatinine, eGFR, and proteinuria. Compared with glomerular PLA2R deposition, serum anti-PLA2R antibody levels are more closely correlated with disease activity and renal function (32). Serum anti-PLA2R antibody levels are detectable months to years before proteinuria production (33) and decline or disappear early before immune deposits start to recede (34). In addition, proteinuria levels are higher in patients with anti-PLA2R antibody-positive IMN than in patients with anti-PLA2R antibody-negative IMN (26, 35), and immunosuppressive therapy is more effective against PLA2R antibody-negative IMN than against PLA2R-positive IMN (29). When the anti-PLA2R level is <40 IU/ml, patients are expected to benefit from conservative treatment (36, 37), avoiding the side effects of immunosuppressive agents. Dong et al. (38) found that the remission rate was lower in the serum anti-PLA2R antibody-positive group than in the PLA2R-negative group, with no significant difference between the PLA2R-positive group and the PLA2R-negative group in renal tissues, suggesting that compared with tissue PLA2R, serum anti-PLA2R antibodies are better correlated with the progression and therapeutic response of IMN. Other studies have shown that spontaneous remission rates are higher in patients with MN with low anti-PLA2R titers than in patients with high anti-PLA2R titers (36, 39). In addition, approximately one-third of patients with ESRD undergo renal transplantation, and 30%–50% of these patients have recurrent MN (40, 41). Increased anti-PLA2R antibodies levels in patients with MN before renal transplantation indicate a risk of recurrence after renal transplantation (42–44). Therefore, to evaluate the treatment response and adjust the treatment strategy, KDIGO 2021 glomerular disease management guidelines (25) recommend evaluation at 3 or 6 months after the start of treatment, and the anti-PLA2R antibodies level should be monitored longitudinally.

### 1.2 THSD7A

At present, there are insufficient data to support the application of anti-THSD7A antibodies as a diagnostic biomarker for MN instead of biopsy (25). Although THSD7A has good specificity for the diagnosis of IMN, its sensitivity is low. A recent meta-analysis of 10 studies involving 4,545 patients showed that the total sensitivity of the anti-THSD7A antibody for the diagnosis of IMN was 4%, the specificity was 99%, the total sensitivity for PLA2R-negative IMN was 8% (6%–10%), and the specificity was 100% (99%–100%), suggesting that anti-THSD7A antibodies have high diagnostic value for PLA2R-negative IMN and can be used as an auxiliary diagnostic method (45).

Similar to the anti-PLA2R antibody, the anti-THSD7A antibody is mostly detected in IMN; however, it cannot completely distinguish IMN from SMN. Maifata et al. (30) detected the serum anti-THSD7A antibody in four patients with IMN (4/47, 8.5%) and in two patients with SMN (2/22, 9.1%). In addition, studies have shown that the anti-THSD7A antibodies levels are associated with the treatment response and disease activity (46). Patients with high anti-THSD7A titer at baseline had poor prognosis (46). Therefore, monitoring the autoantibody titer can contribute to disease monitoring and prediction of curative effects in THSD7A-related MN.

### 1.3 EXT1/EXT2 and NCAM-1

The etiology of SMN is mainly autoimmune diseases, and LN is the main cause of SMN caused by autoimmune diseases. Approximately 30%–40% of SMN are EXT1/EXT2-related MN, and nearly 35% of EXT1/EXT2-related SMN are membranous LN. Therefore, EXT1/EXT2 is considered the main subtype of SMN and can be used as a potential representative biomarker of SMN and LN (12). In addition, a retrospective study has shown that patients with LN who are EXT1/EXT2 positive have fewer chronic renal changes and a lower rate of progression to ESRD than negative patients (47); therefore, EXT1/EXT2 may have predictive value for the prognosis of LN. However, to date, anti-EXT1/EXT2 antibodies have been identified only in renal tissues and not in the peripheral circulation (12), which indicates that they may not be considered to be noninvasive biomarkers for MN diagnosis.

NCAM-1 is another podocyte target antigen in LN. It is found in the tissues of patients with membranous LN, with no positive staining in normal kidney tissues. Studies have shown that approximately 6.6% of NCAM-1 associated renal disease is membranous LN and 2.0% is PMN. Circulating anti-NCAM1 antibodies have been confirmed to exist in a subset of lupus patients (15). Both NCAM-1 and EXT1/EXT2 are considered the first-generation biomarkers of membranous LN (20).

### 1.4 NELL-1, Sema-3B, PCDH7, and HTRA1

In NELL-1-, Sema-3B-, PCDH7-, and HTRA1-related MN, their respective autoantigens and circulating autoantibodies can be identified, which can be considered as potential biomarkers for MN diagnosis. In addition, NELL-1-, Sema-3B-, PCDH7-, and HTRA1-related MN also have their own clinical characteristics, which are helpful for accurate clinical diagnosis and treatment.

One characteristic of NELL-1 associated MN is that malignant tumors may exist in it. The incidence of NELL-1-related MN malignant tumors ranges from 11.7% to 33% (48). MN may also occur before the detection of malignant tumors. Therefore, MN patients who are NELL-1 serum antibody- positive may need to consider regular physical examinations to exclude the presence of malignant tumors.

Sema-3B-associated MN occurs in children and young people. Sethi et al. showed that among 11 cases of Semaphorin 3B-associated MN, 8 were pediatric cases and 5 cases started at or below the age of 2 (14). In pediatric patients, if renal biopsy is not performed, MN may be misdiagnosed. Therefore, if the Sema-3B antibody is detected in children with NS, MN should be considered.

HTRA1 and PCDH7 are newly discovered MN podocyte target antigens based on MS (16, 17). It has been reported that approximately 1%–2% of the target antigens in IMN may be HTRA1 (16) and 5%–6% of the target antigens in PLA2R-MN may be PCDH7 (17). To date, it is not clear whether or not PCDH7-associated MN belongs to IMN. What the characteristic of PCDH7-related MN has been found is that complement activation is rarely activated or not activated, and some patients have spontaneous remission without immunosuppressive therapy (17); however, further clinical studies are needed to determine whether PCDH7 can be used as a biomarker of spontaneous remission of MN (18).

## 2 MN-Related Non-Podocyte Autoantigen or Autoantibody Biomarkers and Metabolic Biomarkers

Exploring differentially expressed proteins and metabolites between patients with MN and healthy individuals is an important research goal, providing candidate biomarkers and improving our understanding of the underlying molecular mechanisms. Recently, various proteins and metabolic biomarkers have been detected by proteome and metabolome analysis based on MS technologies (**Table 2**).

**Table 2:** Summary of research progress on MN-related non-podocyte target antigen proteins and metabolic biomarkers.

Year	Biomarkers	Sample type	Technique	Clinical value	Reference
2015	LIMP-2	Urine	iTRAQ	Diagnosis	(49)
2017	carbamic acid monoammonium salt, 2-pentanone, 2,4-dimethyl- pentanal, hydrogen azide, thiourea, 4-heptanone	Urine	GC/MS	Diagnosis	(50)
2018	A1AT, AFM	Urine	TMT, LC–MS/MS	Diagnosis	(51)
2018	SAA1	Serum	HPLC–MS	Response to CNI treatment	(52)
2019	α-hydroxybutyric acid, 3,4-dihydroxymandelic acid, 5a-cholestanone, 2-hydroxyglutaric acid lactone, nicotinamide, epicoprostanol, and palmitic acid (a set of metabolites)	Urine	NMR, GC–MS/MS	Diagnosis	(53)
2019	MIF	Tissue	MALD-MSI	Response to immunosuppressive treatment	(54)
2020	Patterns of cationic and acidic urinary albumin species	Urine	CIEF-MS	Differential diagnosis of IMN and SMN	(55)

Differences in the levels of various non-podocyte autoantigen or autoantibody proteins and metabolites have recently been reported between MN and healthy controls or patients with other glomerular diseases. These differential proteins and metabolomics are expected to become potential diagnostic biomarkers for MN. Using the iTRAQ (relative and absolute quantitative isobaric labeling) technique, Root et al. (49) found that the level of lysosomal-integrated membrane protein (LIMP-2) in urinary microbubbles obtained from patients with MN was twofold higher than those in idiopathic focal segmental glomerulosclerosis (FSGS) and normal controls. Wang et al. (50) used gas chromatography/mass spectrometry (GC/MS) to analyze the contents of volatile organic compounds in the urine of 63 patients with IMN and 15 normal controls. The results showed that six volatile organic compounds (carbamic acid monoammonium salt, 2-pentanone, 2,4-dimethyl-pentanal, hydrogen azide, thiourea, and 4- heptanone) differed significantly between patients with IMN and normal controls (p < 0.05). Pang et al. (51) performed two technical replicates (TMT1 and TMT2) by using tandem mass tag (TMT) systems combined with nanoscale LC-MS/MS to analyze and compare the urine proteomes of PMN patients and healthy controls. In total, 509 and 411 proteins were identified in TMT1 and TMT2, respectively, and 249 proteins were identified in two technical replicates, of which urine alpha-1-antitrypsin (A1AT) and afamin (AFM) were oversecreted in patients with PMN. In addition, Taherkhani et al. (53) analyzed the urinary metabolomes of 66 patients with MN, 31 healthy controls, and 72 disease controls by nuclear magnetic resonance (NMR) and GC-MS/MS. A set of seven metabolites (a-hydroxybutyric acid, 3,4-dihydroxymandelic acid, 5a-cholestanone, 2-hydroxyglutaric acid lactone, nicotinamide,epicoprostanol, and palmitic acid) was the best diagnostic predictor of MN. The detection sensitivity and specificity of these seven metabolites were 100%.

Tie et al. (55) used a newly developed the capillary isoelectric focusing-mass spectrometry (CIEF-MS) method to compare the types of urinary albumin in patients with IMN and SMN. Cationic and acidic albumin in the urine patterns differed between IMN and SMN, suggesting that the detection of urinary albumin patterns could be used to distinguish between IMN and SMN.

In addition, Yu et al. (52) used nano- high performance liquid chromatography (HPLC)-MS to screen differential proteins between an IMN remission group and non-remission group after treatment with calcineurin inhibitors (CNI). The level of serum amyloid A1 protein (SAA1) in the remission group was significantly higher than that in the non-remission group, suggesting that SAA1 is a candidate marker to predict the efficacy of calcineurin inhibitor treatment in IMN.

A study of two groups of patients with different responses to immunosuppressive therapy (Ponticelli protocol) had kidney biopsies and performed matrix-assisted laser desorption/ionization mass spectrometry imaging (MALDI-MSI), which revealed that the signal at m/z 1,303 showed the greatest discriminatory power, and the corresponding trypsin peptide was identified as a macrophage migration inhibitory factor (MIF). These results suggest that MIF may be a predictor of the response to immunosuppressive therapy in MN (54).

Although these non-podocyte target antigens and autoantibody biomarkers have not been widely validated and little is known about correlations between these biomarkers and clinical manifestations and pathological features, the discovery of these biomarkers provides a basis and new directions for diagnosis, differential diagnosis, monitoring, and treatment of MN.

## 3 MN-Related ncRNAs

Noncoding RNAs (ncRNAs) are a kind of RNA that do not encode proteins but have biological functions at the RNA level, including ribosomal RNAs, transport RNAs, small nucleolar RNAs, small nucleolar RNAs, long-chain noncoding RNAs (lncRNAs), circular RNAs (circRNAs), microRNAs (miRNAs), and small interfering RNAs. With the development of gene sequencing technology and bioinformatics methods, the biological functions of ncRNAs have become a focus of recent research. Many studies have shown that imbalances in ncRNA expression are related to the occurrence and development of many diseases. Because ncRNAs stably exist in body fluids and can be detected noninvasively, they are a promising new biomarker type for disease prediction. Recent studies have shown that the expression levels of ncRNAs in the urine, blood, and kidneys of patients with MN differ from those in healthy individuals (**Table 3**). Most studies of MN-related ncRNAs are focused on miRNAs, followed by circRNAs and lncRNAs. The discovery of these MN-related ncRNA markers not only has implications for the clinical diagnosis and treatment of MN but also helps to further reveal the mechanism underlying podocyte injury in the occurrence and development of MN.

**Table 3:** Summary of research progress on MN-related ncRNA biomarkers.

Year	ncRNA	Sample Type	Technique	Value in MN	Reference
2014	lncRNA XIST	Urine	qRT-PCR	Diagnosis	(56)
2015	miRNA-186	Renal tissue	qRT-PCR	Podocyte apoptosis	(57)
2017	miRNA-217	Renal tissue, Plasma	qRT-PCR	Diagnosis, Podocyte apoptosis	(58)
2018	miRNA-130a-5p	Renal tissue	qRT-PCR	Podocyte apoptosis	(59)
2019	miRNA-193a	Urine	qRT-PCR	Diagnosis	(60)
2019	miRNA -193a	Renal tissue	qRT-PCR	Podocyte apoptosis	(61)
2019	miRNA-195-5p, miRNA-195-3p, miRNA-328-5p	Urine	miRNA microarray dataset GSE64306	Podocyte apoptosis	(62)
2019	miR-107, miR-423-5p, Let-7a-5p, etc.	Renal tissue	TaqMan Low-Density Arrays, qRT-PCR	Diagnosis	(63)
2019	circ_101319	Peripheral blood	circRNA microarray, qRT-PCR	Diagnosis	(64)
2019	XIST	Renal tissue	qRT-PCR	Podocyte apoptosis	(65)
2020	miR-30c, miR-186	Plasma, PBMC	qRT-PCR	Diagnosis	(66)
2021	miR-106a, miR-19b, miR-17	Serum	qRT-PCR	Diagnosis	(67)
2021	circ_0000524	Renal tissue	qRT-PCR	Podocyte apoptosis	(68)

### 3.1 MN-Related miRNAs

MiRNAs are small noncoding RNAs containing approximately 22 nucleotides that are involved in posttranscriptional gene silencing. In recent years, some studies have shown that abnormal miRNAs play a significant role in the pathogenesis of many kidney diseases (69, 70). However, studies of miRNAs in MN are limited at present. In 2014, the first study on the differentially expressed miRNAs (DEMs) in peripheral blood lymphocytes of patients with MN was reported, in which high-throughput sequencing technology was used to analyze the miRNA expression profiles in peripheral blood lymphocytes (PBMCs) of patients with MN and healthy controls, revealing 326 DEMs between the two groups (71). Since then, many studies have used high-throughput technology to detect DEMs between MN patients and healthy controls (63, 72). Although these DEMs may play an important role in the pathogenesis of MN and are expected to become potential biomarkers, further clinical studies are needed to confirm their clinical values in MN. At present, only a few miRNAs have been identified as potential biomarkers of diagnosis or podocyte apoptosis in MN, which are reviewed in the following paragraphs.

#### 3.1.1 miR-217

Li et al. (58) found that compared with levels in healthy controls, the expression level of miR-217 was significantly downregulated in MN, and upregulation of miR-217 expression *in vitro* could inhibit the expression of the target gene *TNFSF11* and protect podocytes. The downregulation of miR-217 expression can lead to increased levels of the target gene *TNFSF11* and podocyte apoptosis, suggesting that miR-217 participates in podocyte apoptosis by negatively regulating the expression of the target gene *TNFSF11*. In addition, the area under the curve (AUC) for miR-217 for MN diagnosis was 0.941 (95% CI, 0.904–0.979), and the sensitivity and specificity were 88.9% and 75.9%, respectively. These results showed that miR-217 is involved in podocyte apoptosis and has potential diagnostic value in MN.

#### 3.1.2 miR-130a-5p

miR-130a-5p expression was found to be significantly decreased in renal biopsy specimens in MN patients, and miR-130a-5p overexpression by miR-130a-5p agomir strongly alleviated renal injury in MN mice. In addition, miR-130a-5p was downregulated and further increased the expression of the target gene *PLA2R* in the podocyte damage model stimulated by Ang II. All these findings indicated that the decrease in miR-130a-5p expression in MN patients may accelerate podocyte apoptosis by upregulating the expression of *PLA2R*, thereby promoting IMN development (59).

#### 3.1.3 miR-193

Zhang et al. (60) showed upregulated miR-193a expression and downregulated WT1/PODXL expression in patients with IMN. In addition, four indexes, including high miR-193a expression, low WT1/PODXL expression, high proteinuria (>3.79 g/24 h)/serum creatinine (>174.63 μmol/L), and low GFR (≤68.13 ml/min/1.73 m^2^), are important biomarkers of poor renal survival in patients with IMN. The combined detection of miR-193a with PODXL and WT1 can be used as a strategy for diagnosing IMN (AUC = 0.994) and predicting renal survival status (AUC = 0.824). Although this study failed to figure out how miR-193a/WT1/PODXL axis functioned underlying the etiology of IMN, another study (61) established a cationic bovine serum albumin (cBSA)-induced rat model of MN and found that inhibiting miR-193a can inhibit renal injury in rats, and podocyte injury leads to tissue cell apoptosis. Furthermore, *WT1* is a target gene of miR-193a, and the expression of *WT1* increases after the inhibition of miR-193a. Therefore, membrane nephropathy may be improved by inhibiting miR-193a.

#### 3.1.4 miR-195-5P, miR-192-3P, and miR-328-5p

Zhou et al. analyzed the mRNA database GSE73953 (eight MN cases and two healthy controls) and miRNA database GSE64306 (four MN cases and six healthy controls) datasets (62) and found that the interactions among miRNA-195-5P, miRNA-192-3P, and miR-328-5p and their corresponding target genes *PPM1A*, *RAB1A*, and *BRSK1* in peripheral blood and urine may be involved in MN development and podocyte apoptosis. In addition, PPM1A and BRSK1 may be involved in MN by the MAPK-related signaling pathway, while RAB1A may be associated with MN by involving the p53 signaling pathway. However, further investigation is needed to validate the relationship between these miRNAs and their target genes in *in vitro* or *in vivo* experiments.

#### 3.1.5 miR-107, miR-423-5p, Let-7a-5p, etc.

A recent study (63) confirmed 10 miRNAs (miR-107, miR-423-5p, Let-7a-5p, etc.) to be DEMs in renal tissue between MN patients and the control group by quantitative real-time PCR (qRT-PCR). According to Gene Ontology analysis, these 10 miRNAs may regulate pathways of epidermal growth factor receptor (EGFR), platelet activation, transforming growth factor beta receptor (TGFBR), mitotic G2/M transition, Forkhead box O (FoxO), extracellular matrix (ECM) disassembly, and SMAD binding, which are associated with MN pathogenesis, including the cell cycle, proliferation, and apoptosis. In addition, transient silencing of miRNAs (let-7a-5p, let-7c-5p) in A498 cells significantly upregulated *IL-6* and *MYC* which have strong negative correlations with clinical parameters, such as proteinuria and total cholesterol. These data indicated that the 10 miRNAs and their downstream network could be considered as potential diagnostic biomarkers of MN and may be involved in MN pathogenesis.

#### 3.1.6 miR-30, miR-186

Sha et al. (57) showed that miR-186 is significantly downregulated in MN, that its expression is regulated by TLR4, and that miR-186 mimic treatment reversed the effects of apoptosis induced by Angiotensin II in podocytes. Recently, another study predicted that the miR-30 family may participate in the pathogenesis of MN by targeting different genes involved in the TGF-β, Notch1, and p53 signaling pathways based on bioinformatics analysis and a literature review. In addition, miR-30c and miR-186 levels were significantly elevated in PBMC (p = 0.037) and plasma (p = 0.035) samples in MN patients compared with the control group, respectively. Moreover, plasma and PBMC miR-30 levels combined with miR-186 detection can be used as a potential biomarker for NS diagnosis (AUC ≥ 0.72) (66).

#### 3.1.7 miR-106a, miR-19b, and miR-17

Wu et al. (67) showed that compared with levels in the control group, the expression levels of miR-106a, miR-19b, and miR-17 in the IMN group were significantly lower, whereas phosphatase and tensin homolog (PTEN) concentration was increased significantly and negatively correlated with the expression of miR-106a and miR-19b in the IMN group, which suggests that miR-106a, miR-19b, miR-17, and PTEN are involved in the pathogenesis of IMN, although their roles in the pathogenesis of IMN are unclear and need to be further explored. In addition, miR-106a, miR-19b, and miR-17 are potential biomarkers for IMN diagnosis with the AUC values for serum miR-106a, miR-19b, and miR-17 of 0.66 (95% CI, 0.56–0.76), 0.81 (95% CI, 0.73–0.89), and 0.69 (95% CI, 0.59–0.79), respectively.

### 3.2 MN-Related circRNAs

Previous studies have shown that the expression levels of circRNAs in the serum and urine exosomes, peripheral blood, and renal tissues differ between patients with IMN and healthy controls. Jin et al. (64) detected 955 differentially expressed circRNAs (645 upregulated and 310 downregulated) between the peripheral blood of patients with IMN and healthy individuals using circRNA chip technology. Among these, circ_101319 expression was significantly upregulated and effectively predicted IMN (AUC = 0.89). In addition, a coexpression network analysis revealed that circ_101319 may be related to pathways related to IMN *via* various miRNAs, providing novel insights for targeted gene therapy.

A circRNA sequencing analysis of serum and urine exosomes from patients with IMN and healthy controls revealed 89 significantly differentially expressed circRNAs in the serum exosomes, of which 49 were upregulated and 40 were downregulated in IMN. There were 60 significantly differentially expressed circRNAs in the urine exosomes, of which 54 were upregulated and 6 were downregulated in IMN. These differentially expressed circRNAs may be involved in the pathogenesis of MN (73).

Recently, Sun et al. (68) found that circ_0000524 is significantly upregulated in the renal tissues of MN. Circ_0000524 regulates the expression of CXCL16 by sponging miR-500a-5p. Downregulating circ_0000524 can inhibit angiotensin II-induced podocyte apoptosis *via* the circ_0000524/miR-500a-5p/CXCL16 pathway, which may play an important role in podocyte injury in MN.

### 3.3 MN-Related lncRNAs

Huang et al. found that lncRNA XIST is upregulated in renal tubular epithelial cells and glomerular cells of mice with MN, and its expression is positively correlated with the severity of MN. In addition, XIST levels are significantly elevated in the urine of MN mouse model and in patients with glomerulonephritis (including MN), and XIST concentrations in the urine are positively correlated with the severity of MN. Accordingly, urine XIST may be a potential noninvasive biomarker of MN (56). In addition, another recent study demonstrated that the downregulation of XIST could reduce podocyte apoptosis via miRNA-217/TLR4 pathway (65).

## 4 MN-Associated Immune Cell Biomarkers

MN is an autoimmune disease, and immune cells are involved in the occurrence and development of MN. Studies have shown increases in the CD4+/CD8+ T-cell ratio and Th2/Th1 cell ratio in the peripheral blood of patients with IMN (74). Although the CD4+/CD8+ T-cell ratio and Th2/Th1 cell ratio are not good biomarkers for IMN, their increase shows a relative increase in Th2 cells. Th2 cells, as the main group of CD4+T cells, can stimulate B cells to produce IgG4 by secreting cytokines IL-4 and IL-10 (74). In addition, anti-CD20 monoclonal antibody (rituximab) achieved good results in the clinical treatment of MN (75, 76). All these results indicated that IMN may be dominated by humoral immunity. However, peripheral blood B cells, as the main lymphocytes of humoral immunity, are not a reliable biomarker of disease activity and treatment outcomes. A study measured the lymphocyte subsets of 66 patients with clinically diagnosed IMN disease and 40 healthy controls. Compared with healthy subjects, it was found that the number of B lymphocytes in peripheral blood had no correlation with renal function indexes. In addition, although the amount of B-lymphocyte infiltration in IMN patients was higher, it was unrelated to the amount of B-lymphocyte infiltration in peripheral blood (77).

T cells participate in the activation and proliferation of B cells in the occurrence and development of MN, and B cells proliferate and differentiate into plasma cells for autoantibody production. Autoantibodies and complement activation can cause the infiltration of macrophages derived from peripheral blood monocytes. In recent years, some subsets of T cells and monocytes in the peripheral blood of patients with MN have been identified as candidate biomarkers to evaluate the curative effect and progression of MN. The candidate immune cell biomarkers and their potential clinical values in MN are described as follows:

### 4.1 Treg Cells

Treg cells are TCRαβ+Foxp3+CD4+ T cells that are endowed with suppressive activity, and the main role of Treg cells is to control the inflammatory and immune response to self-antigens, thereby preventing autoimmune disease; therefore, the lack of Treg cells may occur in many autoimmune conditions (78, 79). Motavalli et al. (80) found that the proportion of Treg cells in the peripheral blood and the expression of Foxp3 are decreased in IMN. However, Treg cells increase significantly in response to rituximab (81, 82). The percentage of Tregs in patients with a clinical response to rituximab was lower at baseline and increased significantly on day 8 of rituximab treatment but remained unchanged in non-responders and in patients receiving only supportive treatment. Therefore, evaluating Tregs may help to predict the early response to rituximab (82).

### 4.2 ICOS+ Follicular Helper T (Tfh) Cells and PD-1+ Tfh Cells

Tfh cells are an independent CD4+ cell subgroup located in lymphatic follicles and are thought to be critical for the formation and function of germinal center in the generation of memory B cells and long-lived plasma cells. ICOS and PD-1 are the two main extracellular markers expressed by Tfh cells. IL-21, as a key effector of activated Tfh cells, induces B-cell proliferation, mediates the differentiation of activated B cells into plasma cells, and promotes IgM, IgG, and IgA production. Shi et al. (83) found that the ratio of ICOS+/PD-1+ Tfh cells and IL-21+ Tfh cells in the peripheral blood cells of patients with IMN is positively correlated with 24-h urinary protein levels. These results suggest that ICOS+ Tfh cells and PD-1+ Tfh cells are potential biomarkers for evaluating the development of IMN.

### 4.3 CD14+ CD163+ CD206+ M2-Like Monocytes

Monocytes, as progenitors of dendritic cells or macrophages in tissues, are important regulators of the immune response. Human PBMCs can be differentiated into M2 monocytes, which are characterized by CD14+ CD163+. After activation, the expression levels of CD206 and CD115 increase and anti-inflammatory mediators, such as interleukin IL-10, are secreted. Hou et al. (84) found that the level of serum IL-10 in patients with early IMN is significantly higher than that in healthy controls. In addition, the number of CD14+ CD163+ CD206+ M2-like cells in patients with early IMN was positively correlated with the 24- h urinary albumin levels and serum PLA2R. Accordingly, CD14+ CD163+ CD206+ M2- like monocytes are potential markers for the severity of IMN.

## Conclusions and Prospects

MN is one of the main pathological types of NS and the major cause of ESRD. At present, renal biopsy is the gold standard for MN diagnosis. Due to its invasive limitation, exploring new biomarkers in MN has become a research hotspot. In recent years, the discovery of MN biomarkers has advanced substantially since the development of high-throughput MS and gene sequencing technologies. Proteomic, metabolomic, and transcriptomic approaches have been used to evaluate the molecular basis of MN.

A variety of MN podocyte target antigens and their autoantibodies have been discovered, among which the anti-PLA2R antibody provides a good theoretical basis for the diagnosis of clinical IMN. However, of the IMN cases, the percentage of PLA2R-positive MN is approximately 70%–80%, and there are still 10%–20% of IMN-related podocyte target antigens unknown that need to be discovered.

Furthermore, various new biomarker types, such as proteins, metabolic markers, ncRNAs, and immune cells, have recently been shown to have diagnostic, monitoring, and prognostic significance in MN. Although these new markers have not been used in clinical settings and need to be verified, they may guide the development of diagnostic and therapeutic approaches for MN and improve our understanding of the pathogenic mechanism of MN.

## Author Contributions

XQ and QL participated in the conceptual design and wrote the manuscript. QL, JL, BX, YZ, MM, and MY wrote the manuscript and created the figures and tables. XQ, QL and JL revised and edited the manuscript. All authors contributed to the article and approved the submitted version.

## Funding

This study was supported by the National Key Research and Development Program of China (No. 2018YFE0207300). This work was also supported in part by the “345 Talent Project” of Shengjing Hospital of China Medical University (No. 50A) and Major Special Project of Construction Program of China Medical University in 2018 (No. 112/3110118034).

## Conflict of Interest

The authors declare that the research was conducted in the absence of any commercial or financial relationships that could be construed as a potential conflict of interest.

## Publisher’s Note

All claims expressed in this article are solely those of the authors and do not necessarily represent those of their affiliated organizations, or those of the publisher, the editors and the reviewers. Any product that may be evaluated in this article, or claim that may be made by its manufacturer, is not guaranteed or endorsed by the publisher.
